# The Inhibitory Receptor Siglec-8 Interacts With FcεRI and Globally Inhibits Intracellular Signaling in Primary Mast Cells Upon Activation

**DOI:** 10.3389/fimmu.2022.833728

**Published:** 2022-01-28

**Authors:** Wouter Korver, Alan Wong, Simon Gebremeskel, Gian Luca Negri, Julia Schanin, Katherine Chang, John Leung, Zachary Benet, Thuy Luu, Emily C. Brock, Kenneth Luehrsen, Alan Xu, Bradford A. Youngblood

**Affiliations:** ^1^Allakos Inc., Redwood City, CA, United States; ^2^LM Biostat Consulting Inc., Victoria, BC, Canada

**Keywords:** mast cells, IgE receptor, Siglec-8, intracellular signaling, proteomics

## Abstract

Immunomodulation of mast cell (MC) activity is warranted in allergic and inflammatory diseases where MCs have a central role in pathogenesis. Targeting Siglec-8, an inhibitory receptor on MCs and eosinophils, has shown promising activity in preclinical and clinical studies. While the intracellular pathways that regulate Siglec-8 activity in eosinophils have been well studied, the signaling mechanisms that lead to MC inhibition have not been fully elucidated. Here, we evaluate the intracellular signaling pathways of Siglec-8-mediated inhibition in primary MCs using an anti-Siglec-8 monoclonal antibody (mAb). Phospho-proteomic profiling of FcεRI-activated MCs revealed Siglec-8 mAb-treatment globally inhibited proximal and downstream kinases, leading to attenuated MC activation and degranulation. In fact, Siglec-8 was found to directly interact with FcεRI signaling molecules. Siglec-8 inhibition was dependent on both cytoplasmic immunoreceptor tyrosine-based inhibitory motifs (ITIMs) that interact with the SH2 containing protein phosphatase Shp-2 upon Siglec-8 phosphorylation. Taken together, these data support a model in which Siglec-8 regulates proximal FcεRI-induced phosphorylation events through phosphatase recruitment and interaction with FcεRIγ, resulting in global inhibition of MCs upon Siglec-8 mAb engagement.

## Introduction

Mast cells (MCs) are found at the internal and external interfaces of the body, in particular at mucosal surfaces, surrounding blood vessels, and directly interacting with peripheral nerves. They respond to a broad array of both IgE-dependent and -independent activating signals, including allergens, cytokines, complement proteins, Toll-like receptor (TLR) ligands, and neuropeptides (1). Upon stimulation, MCs secrete a broad range of inflammatory mediators that can cause anaphylactic responses and drive acute and chronic inflammation (2). Because of the myriad inflammatory mediators they release, and their ability to recruit and activate other immune cells and respond to their environment, MCs are considered key drivers of pathology in gastrointestinal, ophthalmic, dermatologic, respiratory, and proliferative diseases (3).

Sialic-acid-binding immunoglobulin-like lectin (Siglec)-8 is an inhibitory receptor selectively expressed on human MCs and eosinophils (4, 5). Since Siglec-8 does not have a true functional orthologue in non-primates, studies on receptor function in MCs rely on transgenic mice or *ex vivo* assessments of human cells (6). Targeting Siglec-8 with a monoclonal antibody (mAb) has shown promising inhibitory activity in both pre-clinical and clinical studies (6). Multiple studies have evaluated the intracellular pathways of Siglec-8-mediated cell death in IL-5-primed eosinophils and have demonstrated a role for CD11b/CD18 integrin-mediated adhesion, Src family kinases, Syk, and SHIP1 (7, 8). However, unlike the activity seen in eosinophils, Siglec-8 does not induce death of MCs, suggesting the intracellular pathways of Siglec-8 signaling in eosinophils and MCs are different (9). Like many inhibitory receptors, Siglec-8 contains two immunoreceptor tyrosine-based inhibitory motifs (ITIMs) in its cytoplasmic domain that are thought to participate in negative regulation of MC activation (10, 11). Indeed, Siglec-8 mAbs have been shown to inhibit both IgE-dependent and -independent MC activation *in vivo*, including systemic anaphylaxis in humanized mice and IL-33- and Substance P-mediated MC activation in Siglec-8 transgenic mice (12–14). Similarly, nanoparticles displaying allergen and Siglec-8 ligands have been shown to suppress phosphorylation of kinases activated by high affinity IgE receptor (FcεRI) signaling and reduce IgE-mediated murine anaphylaxis (15). Consistent with Siglec-8 functioning as an inhibitory receptor, the membrane proximal ITIM was shown to be required for Siglec-8 mAb-mediated inhibition of FcεRI-dependent calcium flux and secretory responses in MCs (9). Despite these studies identifying specific components of Siglec-8 signaling in MCs, the intracellular mechanisms that contribute to Siglec-8-mediated inhibition of MCs have not been comprehensively evaluated.

In the current study, we investigate the inhibitory effects of Siglec-8 on FcεRI intracellular signaling in primary MCs using a Siglec-8 mAb. Through phospho-proteome profiling of FcεRI-activated MCs, we find that Siglec-8 mAb treatment globally inhibits intracellular phosphorylation events, including inhibition of the kinases involved in initiating the FcεRI signaling cascade. We demonstrate that both cytoplasmic ITIMs confer Siglec-8 mediated MC inhibition and are required for Shp-2 recruitment. Consistent with broad FcεRI-mediated inhibition, we demonstrate that Siglec-8 directly interacts with FcεRI machinery. Collectively, these data provide additional mechanistic insight into the intracellular signaling pathways that contribute to Siglec-8-mediated MC inhibition.

## Materials and Methods

### Bone Marrow Mast Cell Generation

Bone marrow from the femurs and tibias of wild-type C57BL/6 mice or Siglec-8 transgenic mice (16) was cultured in complete medium (DMEM supplemented with 10% FBS (Cytiva), 2 mM L-glutamine (Gibco), 1% antibiotic-antimycotic solution (Gibco), 50 µM β-mercaptoethanol (Gibco) in the presence of 10ng/mL mIL-3, Stemcell Technologies). After 2 days of culture, cells in suspension were transferred to a new flask and fresh culture medium was added two times per week with the cell count maintained between 0.5-1x10^6^/mL for 6-8 weeks prior to use. MC maturity was monitored using flow cytometry staining with antibodies against CD45 (clone 30-F11, Biolegend), and MC markers CD117 (clone 2B8, eBiosciences) and FcεRIα (clone MAR-1, Biolegend). Siglec-8 expression was monitored using PE conjugated antibody from R&D Systems (clone 837535). Bone marrow mast cells are referred to as BMMC (from C57BL/6 wild-type mice) and S8-BMMC (from Siglec-8 transgenic mice).

Methods for human MC generation and activation can be found in the **Supplementary Material**.

### FcεRI-Mediated Bone Marrow Mast Cell Activation and Siglec-8 mAb Treatment

S8-BMMC were plated in 96-well round bottom tissue culture plates at 5x10^4^ per well and centrifuged for 2 min at 400g prior to resuspending with biotinylated anti-FcεRI (clone MAR-1, Biolegend) at 250ng/mL plus biotinylated isotype-control mouse antibody (MOPC21, mouse IgG1, Allakos) or biotinylated Siglec-8 mAb (2E2, mouse IgG1, Allakos) at 5μg/mL at 4°C for 2 min. Cells were washed in PBS and then incubated in PBS with 10μg/mL neutravidin (Thermo) for 2 min. After an additional PBS wash, cells were resuspended in 200μl 37°C complete medium and incubated for 20 min at 37°C for flow analysis or for 1 or 6 hours for analysis of histamine or cytokine levels in the supernatant. For flow cytometry, cells were resuspended in 100μl cold FACS buffer (PBS/1%BSA) containing 100ng anti-CD63-PE/Cy7 antibody (clone NVG-2, Biolegend), 100ng anti-CD107a-PE (clone 1D4B, Biolegend), 3μl 7-AAD (Becton Dickinson) as viability marker and 0.2μl mouse Fc block (BD). The percent of CD63 and CD107a expressing cells was determined by flow cytometry on a Novocyte Quanteon (Agilent). For cytokine quantification, 25μl of supernatant was analyzed using Meso Scale Discovery’s U-plex kit (customized for the quantification of the indicated cytokines). Histamine levels were determined using an enzyme immunoassay kit (Beckman Coulter).

### Transfections, Immunoprecipitation and Western Blotting

BMMCs (2x10^6^) were transfected with 10μg plasmid expressing wild-type or mutant full-length Siglec-8 fused with an N-terminal FLAG tag. In addition to the wild-type sequence, plasmids containing ITIM mutants of Siglec-8 were generated by changing the tyrosine residue at position 447 of the proximal motif (wild-type=LHYATL) to phenylalanine (Y447F=LHFATL) and the tyrosine at position 470 of the distal motif (wild-type=SEYSEIK) to phenylalanine (Y470F= SEFSEIK). The double mutant contains both mutations (Y447F+Y470F). For all transfections, plasmid DNA (10 μg) was added to 2x10^6^ cells and transfected using the 4D-Nucleofector (Lonza) with P3 nucleofector solution and Supplement 1 and program DS-130. Transfection efficiency of BMMC was consistently between 80-95% under these conditions. Cells were incubated at 37°C in complete medium for 6 hours for lysis for IP/WB or 16 hours for functional assays (FcεRI cross-linking). A fresh 30mM stock solution of pervanadate was prepared by adding 150μl of 200mM sodium orthovanadate (FivePhoton/Fisher) to 844μl PBS, plus 6.1μl 30% H_2_O_2_ (Sigma) and incubated for 20 min at room temperature prior to addition to cells at the indicated final concentrations to inhibit phosphatases.

Cells were lysed in Pierce IP lysis buffer (Thermo) with HALT protease and phosphatase inhibitors (Thermo). Lysates were used directly for Western Blotting or immunoprecipitation by adding 25μl Pierce anti-FLAG or anti-HA magnetic agarose beads (Thermo) for 30 min at room temperature while rotating. Siglec-8 IPs were performed using anti-Siglec-8 polyclonal rabbit antibody (Thermo, PA5-28846) at 10μg/mL for 30 min followed by addition of Magnetic Protein A/G beads (Pierce). After 4 washes with TBS-T, proteins were eluted by incubation at 70°C for 10 min in 1xLB with reducing agent added (Thermo), separated by SDS-PAGE and subjected to Western Blot analysis. 5% milk (Bio-Rad) in TBS-0.1%Tween-20 was used for blocking. Anti-FLAG antibody was from Sigma; anti-HA, anti-Lyn (clone LYN-01) and anti-Siglec-8 from Thermo; anti-Shp-2 (clone D50F2) from Cell Signaling Technologies; 4G10 from Millipore/Sigma. Anti-FLAG and anti-4G10 antibodies for Western blotting were directly conjugated to HRP, other primary antibodies were detected using HRP conjugated mouse anti-rabbit IgG, light chain specific (Jackson ImmunoResearch 211-032-171) at 1:2,000 dilution in TBS-T/5% milk. Western blots were developed using Immobilon Forte western HRP substrate (Millipore) and imaged using the iBright imaging system (Thermo).

### Mass Spectrometry

Detailed methods for MS and proteomics analysis can be found in the **Supplementary Material**.

### Confocal Microscopy

S8-BMMC were stained in PBS/1% BSA on ice for 30 min with anti-S8-AF647 (Allakos), anti-FcεRI-PE (clone MAR-1, Biolegend) and fixed in 2% PFA. For crosslinking, S8-BMMC were treated as described under ‘FceRI-Mediated Bone Marrow Mast Cell Activation and Siglec-8 mAb Treatment’ and subsequently fixed in 2% PFA. Fixed cells were spun onto poly-D-lysine (50μg/mL for 1 hour, GIBCO) coated 96-well glass plates (Cellvis) at 2,000g for 5 min and covered in 200ul PBS with 1μg/mL DAPI for nuclear staining. Cells were imaged on a Leica Stellaris 5 confocal microscope using a 63x objective with oil immersion. Images were analyzed using ImageJ. Briefly, cells were identified manually as ROIs. Within ROIs, bright foci greater than 0.6 μm in diameter were defined as punctae. Intensity of PE was then determined by averaging the fluorescent intensity within each punctae mask.

## Results

### Bone Marrow Mast Cells From Transgenic Mice Express Functional Siglec-8 and Respond to FcεRI-Mediated Activation

**Figure 1:**
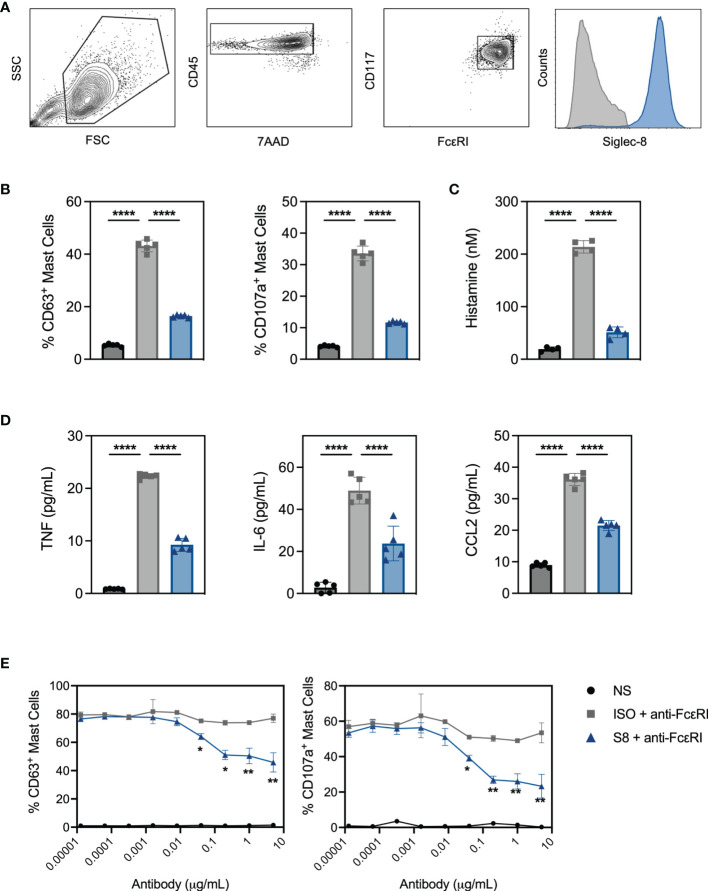
BMMC from transgenic mice respond to FcεRI activation and express functional Siglec-8. **(A)** BMMC-S8 flow cytometry gating strategy. Right panel represents Siglec-8 staining (blue) and FMO stain (grey) **(B)** Levels of surrogate degranulation markers CD63 and CD107a on the cell surface of unstimulated MC (NS; black bars), when cross-linked with the anti-FcεRI antibody MAR-1 for 15 min (ISO + anti-FcεRI, grey bars) or co-cross-linked with MAR-1 and Siglec-8 mAb (S8 + anti-FcεRI, blue bars). **(C)** Histamine concentrations in the culture medium of MC collected 1 hour after stimulation as in **(B)**. **(D)** Cytokine concentrations in the culture medium of MC collected 6 hours after stimulation. **(E)** Titration of isotype control mAb and Siglec-8 mAb in combination with anti-FcεRI-mediated (MAR-1) activation. Left panel: % of CD63 positive MC. Right panel: % of CD107a positive MC. Data are plotted as mean ± SEM (n=5) and are representative of 5 experiments. *p < 0.05, **p < 0.01, ****p < 0.0001 by one-way ANOVA test **(B–D)** or unpaired t test **(E)**.

To interrogate MC activation and inhibition *in vitro*, we developed a system using MCs derived from the bone marrow of Siglec-8 transgenic mice (S8-BMMCs) (16). Upon differentiation after about 6 weeks of culture, these MCs displayed a mature phenotype, including expression of MC phenotypic markers and Siglec-8 (**Figure 1A**).

Stimulation of S8-BMMCs with an agonistic anti-FcεRI antibody in the presence of an isotype control antibody (ISO), induced degranulation as detected by the upregulation of degranulation markers CD63 and CD107a on mast cells (**Figure 1B**). In addition, FcεRI-mediated activation significantly induced histamine secretion (**Figure 1C**, measured after 1 hour), and TNFα, IL-6 and CCL2 cytokine secretion (**Figure 1D**, at 6 hours) compared to unstimulated cells. In the presence of a Siglec-8 mAb, both degranulation and mediator release by activated S8-BMMCs were strongly inhibited compared to isotype control treatment (**Figures 1B–D**). Siglec-8 mAb-mediated inhibition of MC degranulation was concentration dependent: a statistically significant reduction in the upregulation of CD63 and CD107a compared to isotype control treatment was observed with Siglec-8 mAb concentrations of 80ng/mL and higher (**Figure 1E**). To determine if the presence of FcεRI-bound IgE affected Siglec-8 mAb activity, we cultured S8-BMMCs in the presence of titrating concentrations of IgE. While the level of FcεRI-mediated activation was lower in the presence of a high IgE concentration, the inhibition by Siglec-8 mAb was not affected (**Supplementary Figure 1A**). These data demonstrate that S8-BMMCs respond to FcεRI-mediated activation *in vitro* and express functional Siglec-8. To confirm natively expressed Siglec-8 functioned similarly, we evaluated Siglec-8 mAb-mediated inhibition in human primary MCs derived from peripheral blood CD34^+^ cells activated with agonistic anti-human FcεRI antibody CRA-1. Similar levels of Siglec-8 mAb-inhibition were seen in human MCs compared to S8-BMMCs, demonstrating transgenic expression of Siglec-8 in BMMCs is reflective of human function (**Supplementary Figures 1B, C**).

### Siglec-8 mAb Treatment Globally Inhibits FcεRI-Induced Intracellular Signaling

**Figure 2:**
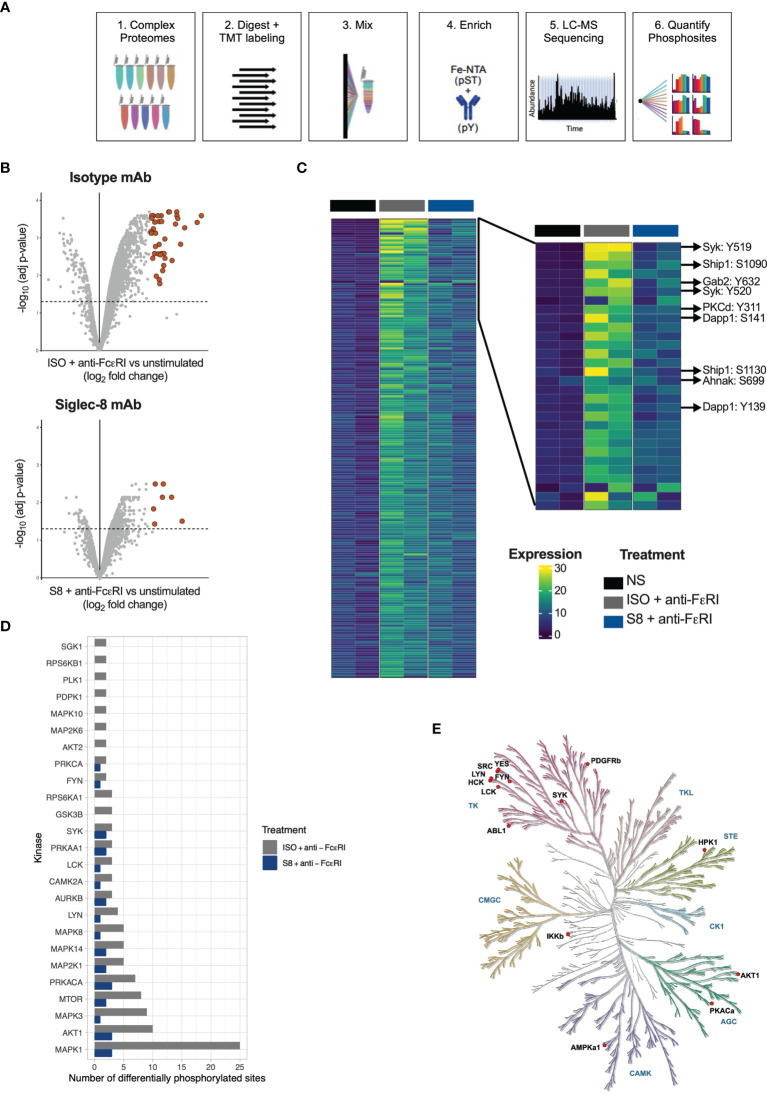
Phospho-proteome of FcεRI-stimulated and Siglec-8-inhibited S8-BMMCs. **(A)** Schematic of the process for determining phospho-proteomes of MCs. **(B)** Volcano plots of Log2 fold change against statistical significance of the abundance of phospho-peptides 2 min after FcεRI crosslinking in the presence of isotype control antibody (top panel) or Siglec-8 mAb (bottom panel). Highlighted are the peptides upregulated more than 4-fold. **(C)** Heatmap of quantified phospho-peptides. Each line represents a unique phospho-peptide with the first two columns representing the unstimulated MCs in two independent experiments, the next two columns the phospho-proteome from MCs activated through FcεRI and the last two columns represent activation in the presence of a Siglec-8 mAb. The heatmap is ranked from top to bottom by fold induction upon activation averaged over the two experiments. Right panel: zoom in on the top sections of the heatmap in **(C)**. **(D)** Bar graph representing the number of phosphosites predicted to be phosphorylated by the indicated kinase in the FcεRI stimulated conditions. **(E)** Kinase tree highlighting the kinases that are known to modify sites that showed differential phosphorylation between the isotype and Siglec-8 mAb conditions upon FcεRI activation.

Inhibition of MCs through Siglec-8 has been described previously (9, 12, 13, 16), but the intracellular signaling pathways have not been fully elucidated. To investigate this, we profiled phospho-proteomes of S8-BMMCs that were stimulated in the absence or presence of Siglec-8 mAb by mass spectrometry. Three treatment conditions were evaluated in duplicate: 1) unstimulated S8-BMMCs, and S8-BMMCs activated by cross-linking of the FcεR1 receptor for 2 min in the presence 2) or absence 3) of Siglec-8 mAb. Proteomes were labeled by Tandem Mass Tag (TMT) and enriched for phospho-serine, -threonine and -tyrosine peptides prior to analysis by mass spectrometry and subsequent quantification of phospho-peptides (**Figure 2A**).

In the FcεRI stimulated MCs, 348 unique phosphosites were identified as differentially phosphorylated by more than 2-fold, of which 41 were upregulated more than 4-fold relative to unstimulated control MCs (adjusted p-value < 0.05; **Figure 2B**). Strikingly, in the presence of a Siglec-8 mAb the number of differential phosphosites decreased to 71 (2-fold) and 7 (4-fold), respectively. A heatmap representing differential phosphorylation events further confirmed that the majority of the FcεRI-induced phosphorylation events were reduced in the presence of Siglec-8 mAb (**Figure 2C**). Many of the abundantly phosphorylated peptides that were decreased represented key proteins known to be immediately downstream of FcεRI signaling (17–19), including the kinase Syk, adaptor molecules Gab2 and Dapp1, and the more downstream kinases protein kinase C and Akt (**Figure 2C**). In addition, Siglec-8 mAb-treatment inhibited signaling molecules phosphorylated upon activation such as Erk, the phosphatase Ship1, scaffold proteins Wipf1, Ahnak and Lat2 and the gamma subunit of FcεRI itself (**Figure 2C** and **Supplementary Figure 2**). The induction and inhibition of global p-Tyr phosphorylation (**Supplementary Figure 3A**) and specific phosphorylation events for p-Syk and p-Erk were confirmed by Western blot and intracellular flow cytometry analysis (**Supplementary Figures 3B–D**).

### Siglec-8 Inhibits Proximal Kinase Activity Upon FcεRI-Mediated Mast Cell Activation

Using the integrative PhosphoSitePlus database (20), we mapped phosphorylation sites to annotated kinases to identify differences in kinase activity between Siglec-8 mAb *vs* isotype conditions. We identified proximal kinases known to be associated with FcεRI activation (Lyn, Syk, Fyn) as well as kinases involved in downstream pathways (e.g., MAPK family members) (**Figure 2D**). FcεRI-activated MCs treated with a Siglec-8 mAb had fewer phosphorylated sites for all predicted kinases compared to activated MCs treated with an isotype control antibody (**Figure 2D**). The kinase tree in **Figure 2E** highlights the kinases known to modify the sites that showed differential phosphorylation (1.2 fold) between isotype and Siglec-8 mAb conditions, including the tyrosine kinases that function at the top of the FcεRI signaling cascade (Syk, Lyn) as well as those downstream (Ikkb). These data demonstrate that Siglec-8 mediates global inhibition of FcεRI signaling in primary MCs by regulating FcεRI-mediated kinase activity upon mAb engagement.

### Both Siglec-8 ITIMs Are Involved in the Inhibition of FcεRI-Induced Degranulation and Phosphatase Recruitment

**Figure 3:**
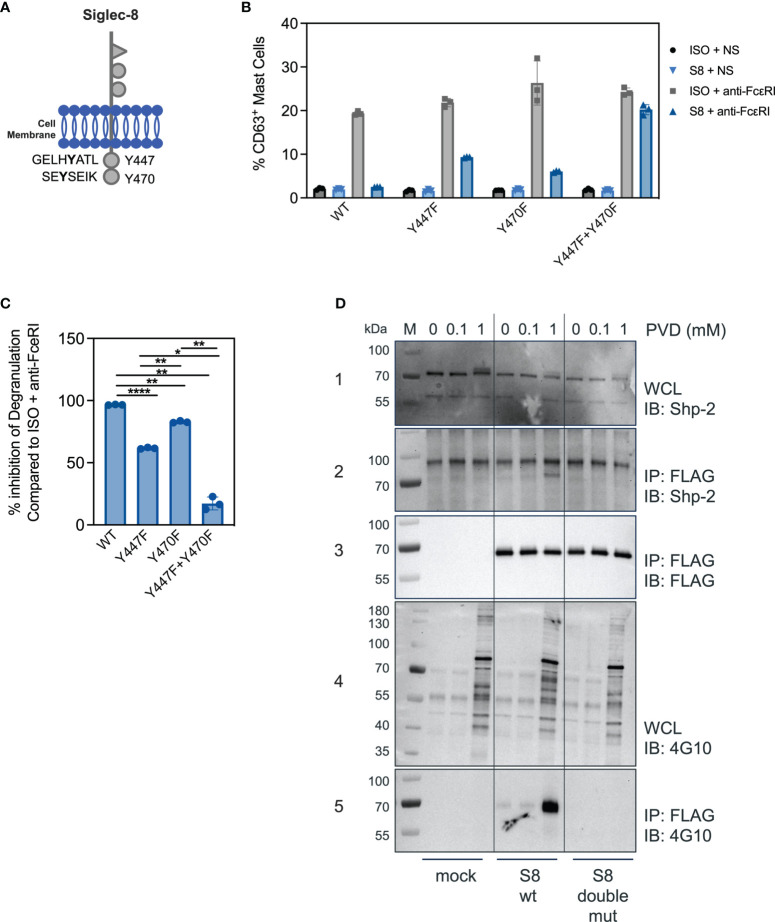
Both ITIM motifs are required for Siglec-8 mediated inhibition. **(A)** Schematic of the Siglec-8 receptor with its two tyrosine residues in the context of ITIM motifs indicated. **(B)** Percent of CD63 positive MC in unstimulated (ISO + NS, S8 + NS), stimulated (ISO + anti-FcεRI) and inhibited (S8 + anti-FcεRI) MC after transfection of the indicated wild-type or mutant Siglec-8 expression constructs Data are plotted as mean ± SEM (n=3) and are representative of 3 experiments. **(C)** Percent inhibition of Siglec-8 mAb-treatment for each Siglec-8 expression construct was calculated compared to ISO condition. *p < 0.05, **p < 0.01, ****p < 0.0001 by one-way ANOVA test. **(D)** Western blot analysis of Siglec-8-Shp2 interaction. Immunoblot (IB) of whole cell lysates (WCL) or FLAG immunoprecipitations (IP) from primary MC mock transfected or with expression constructs for wild-type Siglec-8-FLAG or ITIM double mutant Siglec-8-FLAG. Cells were subjected to PVD treatment for two min and WCL were analyzed for presence of Shp-2 (panel 1) and p-Tyr (panel 4). Anti-FLAG IPs were analyzed for presence of Shp-2 (panel 2), Siglec-8-FLAG (panel 3) and p-Tyr (panel 5). Experiments were performed 2-3 times for confirmation.

Siglec-8 contains two ITIMs in its cytosolic tail (**Figure 3A**). The motif nearest to the membrane was previously demonstrated to be required for Siglec-8 mediated inhibition in rat basophilic leukemia cell activation (9), but whether a single or both tyrosines in the ITIMs of the intracellular domain of Siglec-8 are critical for inhibitory activity in primary MC is unknown. Expression constructs were generated for mutant versions of Siglec-8, in which the tyrosine residues were replaced with phenylalanine residues, either separately (Y447F and Y470F) or combined to generate a double mutant (Y447F+Y470F). BMMCs transfected with each of the wild-type or mutant Siglec-8 constructs were activated *via* anti-FcεRI agonist antibody MAR-1 (ISO+anti-FcεRI). FcεRI activation was inhibited in the presence of a Siglec-8 mAb for BMMCs transfected with wild-type or single-mutant Siglec-8 (**Figure 3B**), although inhibitory function was partially attenuated in BMMCs transfected with the membrane proximal ITIM mutant (Y447F), but not the membrane distal ITIM mutant (Y470F). However, BMMCs expressing the double ITIM mutant lost almost all Siglec-8-mediated inhibition. The percent inhibition calculated from these data obtained for each Siglec-8 variant is plotted in **Figure 3C**. These data demonstrate that both functional ITIMs are required for Siglec-8 mediated inhibition in FcεRI-activated primary BMMCs.

Src-homology 2 (SH2) domain containing protein tyrosine phosphatases have been shown to be critical for inhibition of immune cells through Siglec family members and other ITIM containing receptors (10, 21). We detected Shp-1 at low levels in BMMCs and did not observe an interaction with Siglec-8 (data not shown). In T cells, mechanistic and single cell imaging studies of programmed cell death 1 (PD-1) demonstrated that Shp-2 is required for inhibition (22, 23). Since the ITIMs were required for Siglec-8 mediated inhibition in BMMCs (**Figure 3B**), we assessed if Shp-2 physically interacted with Siglec-8 in BMMCs transfected with FLAG-tagged Siglec-8 (wild-type or double-mutant) in the presence or absence of the phosphatase inhibitor pervanadate (PVD). In the absence or in the presence of 0.1mM concentrations of PVD, Shp-2 did not co-immunoprecipitate with Siglec-8 despite the presence of Shp-2 protein in whole cell lysates under all conditions (**Figure 3D**, panel 1 + 2). Incubation with a high concentration of PVD (1mM) resulted in Shp-2 co-immunoprecipitating with wild-type Siglec-8, but not with the double ITIM mutant (**Figure 3D**, panels 2 + 3). To evaluate if the Shp-2/Siglec-8 interaction was dependent on Siglec-8 ITIM phosphorylation, we probed with a pan-tyrosine phosphorylation antibody (**Figure 3D**, panel 4). Indeed, wild-type but not the double ITIM mutant Siglec-8 was tyrosine phosphorylated at the high PVD concentration (**Figure 3D**, panel 5). These data strongly suggest that Siglec-8 phosphorylation within the ITIM motifs is required for Shp-2 interaction.

### Siglec-8 Interacts With Proximal FcεRI Signaling Machinery

**Figure 4:**
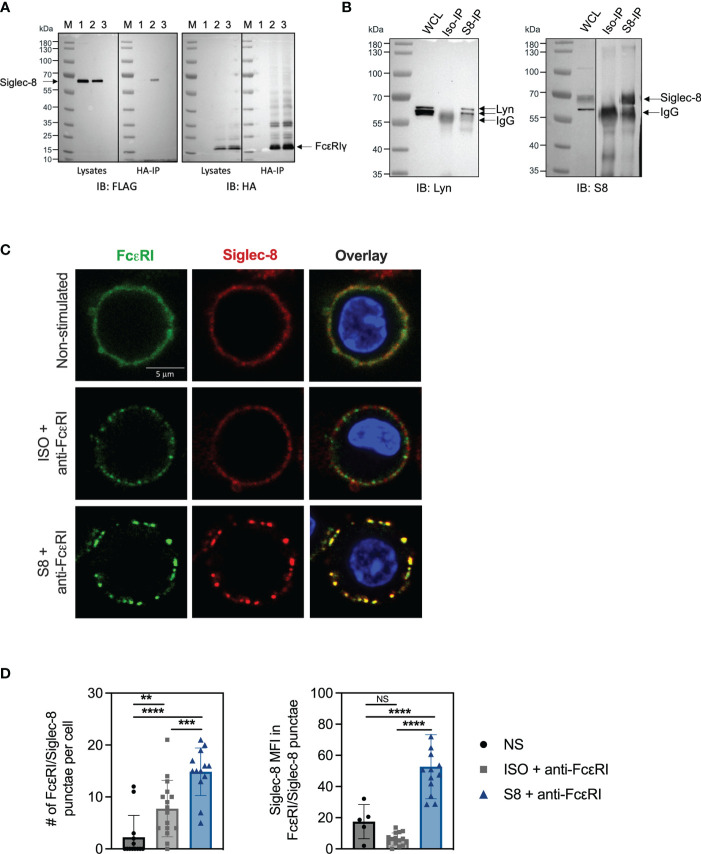
Siglec-8 interacts with FcεRI machinery components. **(A)** Western blot analysis of Siglec-8-FcεRIγ interaction. Immunoblot (IB) of whole cell lysates (WCL) or HA immunoprecipitations (IP) from primary MC transfected with expression constructs for Siglec-8-FLAG (lane 1) or FcεRIγ-HA (lane 3) or both (lane 2). Blots were developed with anti-FLAG (left panel) and anti-HA (right panel). **(B)** Western blot analysis of Siglec-8-Lyn interaction. WCL, isotype IP or S8 IP from S8-BMMC were developed using anti-Lyn or anti-Siglec-8 antibodies. **(C)** Confocal microscopy imaging of Siglec-8 (red) and FcεRI (green) in S8-BMMC. **(D)** Quantification of confocal images. Left panel: number of FcεRI punctae per cell (PE channel). Right panel: Siglec-8 median fluorescence intensity within FcεRI/Siglec-8 punctae (AF647 channel). Experiments were performed 2-3 times. NS, not significant, **p < 0.01, ***p < 0.001, ****p < 0.0001 by one-way ANOVA test.

The inhibition of FcεRI-mediated proximal signaling events led us to hypothesize that Siglec-8 may directly interact with FcεRI signaling components. We investigated potential physical interactions of Siglec-8 with FcεRI and associated signaling molecules using BMMCs transfected with FLAG-tagged Siglec-8. Surprisingly, Siglec-8 co-immunoprecipitated with FcεRIγ from lysates of unstimulated MC, demonstrating that these proteins can co-exist in a complex (**Figure 4A**). Since the Src kinase Lyn is immediately activated upon IgE cross-linking of FcεRI (24–26), and its predicted level of phosphorylation was decreased in Siglec-8 mAb-treated MCs (**Figures 2D, E**), we evaluated if Lyn also interacted with Siglec-8. Indeed, we detected Lyn in the immunoprecipitate of Siglec-8, demonstrating that Lyn also can interact in complex with Siglec-8 (**Figure 4B**).

**Figure 5:**
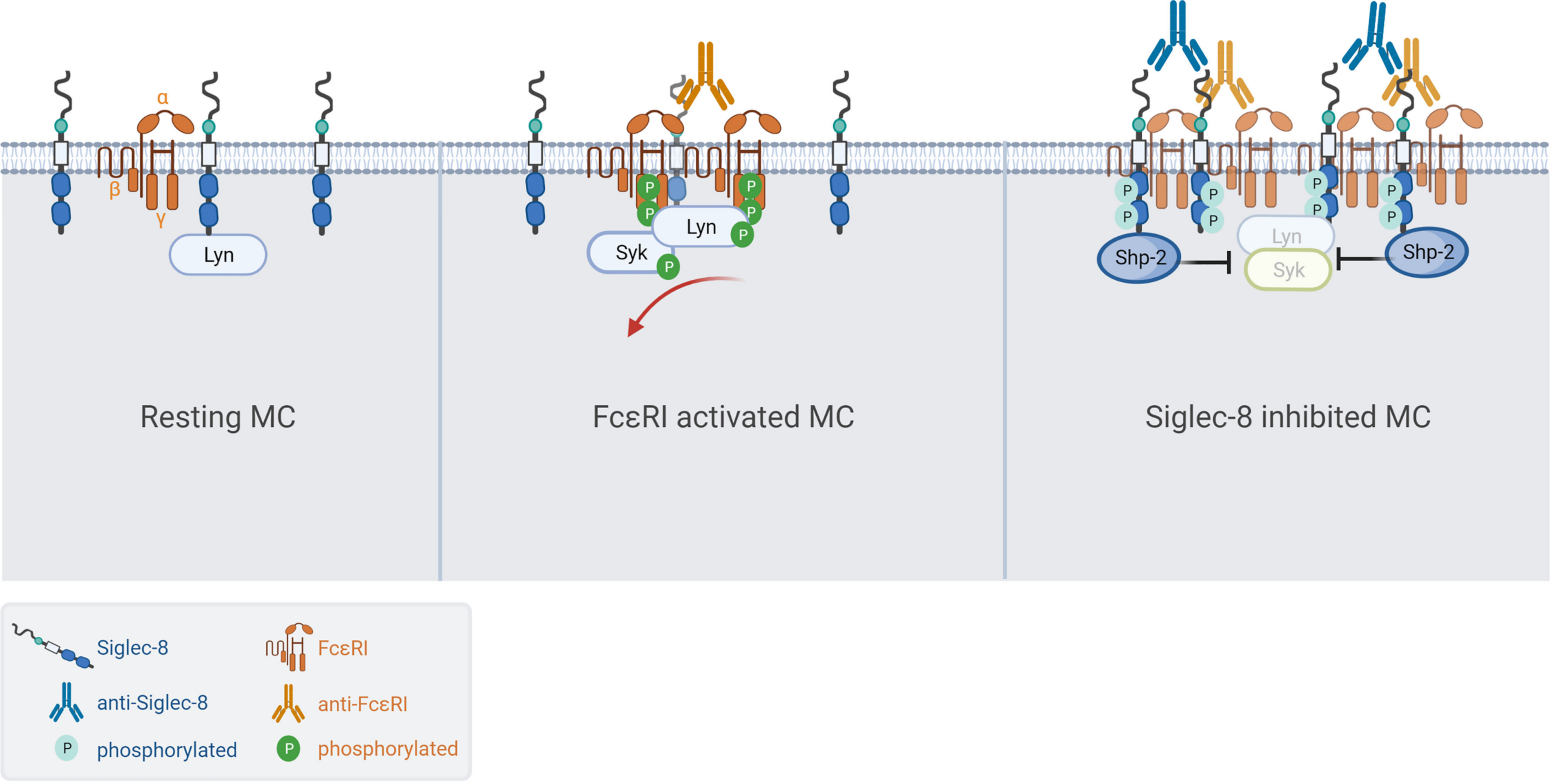
Model of Siglec-8 mediated inhibition of FcεRI signaling in MCs. (Left) Siglec-8 and FcεRI are distributed along the membrane of resting MC while partially colocalizing. (Middle) Cross-linking with MAR-1 anti-FcεRI results in high density clusters of FcεRI resulting in phosphorylation at the ITAM, subsequent MC activation/degranulation and partial exclusion of Siglec-8 from the activating complexes. (Right) In the presence of Siglec-8 mAb, phosphatases like Shp-2 are recruited to large complexes of activating (FcεRI) and inhibitory (Siglec-8) receptors resulting in potent inhibition of intracellular signaling.

Next, we investigated if Siglec-8 colocalized with FcεRI in S8-BMMCs using confocal microscopy. We observed a relatively homogeneous distribution of Siglec-8 and the FcεRI receptor complex on the membrane of unstimulated S8-BMMCs, suggesting they may partially co-localize, in line with the immunoprecipitation data (**Figure 4C**, top panels). Co-cross-linking of both FcεRI and Siglec-8 resulted in large membrane associated structures (punctae) (**Figure 4C**, bottom panels), in which Siglec-8 and FcεRI co-resided. Interestingly, co-localization of both receptors was reduced when only FcεRI was engaged with mAb (**Figure 4C**, middle panels). Quantification of the confocal images confirmed a significant increase in the number of punctae and the presence of Siglec-8 within these punctae when FcεRI and Siglec-8 were both co-cross-linked compared to unstimulated or FcεRI only engagement (**Figure 4D**). The level of Siglec-8 present in complex with FcεRI was trending lower when FcεRI was crosslinked by itself compared to unstimulated cells. Taken together, these data demonstrate that Siglec-8 interacts with FcεRI and its signaling components and suggest the existence of multi-protein complexes where activating and inhibitory receptors interact with multiple proximal signaling mediators. We propose a model whereby recruitment of sufficient levels of Siglec-8 to FcεRI complexes results in increased phosphatase activity that inhibits proximal kinase activity leading to broad attenuation of intracellular signaling and subsequent degranulation in MCs (**Figure 5**).

## Discussion

In recent years, our understanding of the interplay between activating and inhibitory receptors in immune cells has greatly increased through large scale expression and proteomics studies. As key effector cells in acute and chronic allergic and inflammatory responses, MC numbers and activation state have been shown to be aberrantly increased in several diseases (3, 27, 28). The inhibitory receptor Siglec-8 has emerged as a promising drug target in MC-driven diseases and the work described herein was carried out to study in detail the effects of Siglec-8 engagement on intracellular signaling upon MC activation through FcεRI.

While inhibition of FcεRI-mediated activation of MC through Siglec-8 engagement has been demonstrated previously (9, 15, 16), the consequences of this inhibition on intracellular signaling have not been thoroughly investigated. Here we present the first comprehensive evaluation of phospho-proteomes from FcεRI-activated and Siglec-8-engaged MCs. Our data support the conclusion that broad inhibition of proximal anti-FcεRI-induced phosphorylation events in primary MCs results in attenuated degranulation and mediator release in the presence of Siglec-8 mAb.

The inhibition by a Siglec-8 mAb is contingent on the presence of intracellular tyrosine residues within the ITIM domains, as has been described for other Siglec family members (9, 29, 30). We provide evidence that both ITIM domains in Siglec-8 participate in inhibiting FcεRI-induced activation in primary S8-BMMCs. This is analogous to the observed requirement for inhibition through CD22 (Siglec-2) in B cells, which was shown to depend on all three of its ITIMs for fully efficient inhibition (30). In contrast, data from studies with Siglec-7 and Siglec-9 demonstrated only the most proximal of two ITIMs to be critical for inhibitory activity (29). The differences between the requirements of ITIM motifs for inhibition may depend on 1) the origin of the cell type used, whether it is a primary cell or immortalized line, 2) the expression of downstream signaling molecules in these cells, particularly SH2 containing protein tyrosine phosphatases Shp-1 and Shp-2 and 3) the conformation/dimerization of the receptors. In structural studies, Shp-2 was found to require both of its SH2 domains for binding to phosphorylated ITIMs (31), which may be achieved by engaging with two motifs on the same receptor or through dimerization of receptors. While Shp-1 was detected at low levels in BMMCs, we cannot rule out a role for Shp-1 similar to Shp-2 based on our data.

In contrast to the cell death-inducing activity mediated by Siglec-8 mAb antibodies in eosinophils (12, 32), our *in vitro* assessments of Siglec-8 mediated MC activity revealed little evidence of inhibition by a Siglec-8 mAb in the absence of FcεRI-induced activation. These data suggest that a close physical association of activating and inhibitory receptors is required for inhibition and are consistent with recent work studying CD33 (Siglec-3) and Siglec-8 (15, 33). The authors demonstrate that FcεRI and Siglec-8/CD33 are not entirely co-localized in the same membrane microdomain and active recruitment of the inhibitory receptor to the lipid rafts containing the IgE-FcεRI complex is required. However, Siglec-8 mAbs have also been shown to inhibit non-ITAM-mediated activation of MCs, including stimulation through cytokine receptors, GPCRs, and TLRs (13, 14, 28). These data suggest that Siglec-8 could modulate intracellular signaling pathways differently depending on the mode of MC activation. In support of this, transcriptomic profiling of peritoneal MCs stimulated with IL-33 and treated with a Siglec-8 mAb showed significantly increased expression of genes associated with TLR inhibition, MyD88 signaling, and other ITIM-containing receptors (13). Additional studies are needed to elucidate how Siglec-8 and its engagement inhibits IgE-independent MC activation.

Interaction of Siglec-8 with FcεRIγ was detected in co-immunoprecipitation and imaging experiments (**Figures 4A, C****)**, which suggests that at least subsets of these two proteins are in the same protein complex in resting MC. Recent high precision microscopy studies demonstrated, in accordance with our model, subtle changes in location and organization in the plasma membrane between resting and stimulated states, with lipid-based and protein-based interactions leading to suprathreshold phosphorylation by facilitating access of kinase and exclusion of phosphatase activity (34). Collectively, our data provide novel mechanistic insight into intracellular signaling pathways that contribute to Siglec-8 mediated MC inhibition and suggest that Siglec-8 can influence the complex orchestration of activating and inhibitory activities by regulating upstream kinases and phosphatases upon stimulation through FcεRI.

## Data Availability Statement

The original contributions presented in the study are publicly available. This data can be found here: ProteomeXchange, PXD030820 link: http://proteomecentral.proteomexchange.org/cgi/GetDataset?ID=PXD030820

## Ethics Statement

Ethical review and approval was not required for the animal study because the involvement of animals was solely for the harvesting of cells for culturing and *in vitro* experiments. This study was conducted in accordance with the ethical conduct in the care and use of animals and in compliance with ARRIVE guidelines.

## Author Contributions

WK, JS, BY designed experiments. WK, AW, SG, JS, KC, JL, ZB, TL, EB, KL conducted experiments. GN analyzed proteomics data and provided bioinformatics support. WK, BY wrote the manuscript. All authors contributed to manuscript revision, read, and approved the submitted version.

## Conflict of Interest

WK, AW, SG, JS, KC, JL, ZB, TL, EB, KL, AX and BY were employed by Allakos Inc. GN was employed by LM Biostat Consulting Inc.

The authors declare that this study received funding from Allakos Inc. The funder had the following involvement with the study: data collection, analysis, interpretation, study design, and writing of this article.

## Publisher’s Note

All claims expressed in this article are solely those of the authors and do not necessarily represent those of their affiliated organizations, or those of the publisher, the editors and the reviewers. Any product that may be evaluated in this article, or claim that may be made by its manufacturer, is not guaranteed or endorsed by the publisher.
