# Supplementary material for: The Inhibitory Receptor Siglec-8 Interacts With FcεRI and Globally Inhibits Intracellular Signaling in Primary Mast Cells Upon Activation
Source: Front Immunol. 2022 Jan 28;13:833728. doi: 10.3389/fimmu.2022.833728 (PMC8837033; doi:10.3389/fimmu.2022.833728)
Supplement: Supplementary file 1 [file DataSheet_1.docx]

Supplementary Material

# Supplementary Data

## Supplementary Methods

### Generation of human MC (huMC) from CD34+ cells

### Human MC were generated from CD34+ hematopoietic stem cells isolated from whole blood as described (1).

### FcεRI-mediated huMC activation and Siglec-8 mAb treatment

Cell were plated in 96-well round bottom tissue culture plates at 1.5x10^4^ per well and centrifuged for 2 min at 400g prior to resuspending in biotinylated anti-FcεRI (clone CRA-1, Biolegend) plus biotinylated isotype antibody (MOPC21, Allakos) or Siglec-8 mAb (2E2, Allakos) at 5μg/mL at 4 ⁰C for 2 min. Cells were washed in PBS and then incubated in PBS with 5μg/mL secondary antibody (Goat anti-mouse IgG light chain specific, Jackson Immunoresearch) for 2 min. After an additional PBS wash, cells were resuspended in warm complete medium and incubated for 20 min at 37 ⁰C. For flow cytometry, each well was resuspended in 100μl cold FACS buffer (PBS/1%BSA) containing 5μl anti-CD63-PE/Cy7 antibody (clone H5C6, Biolegend), 5μl anti-CD107a-PE (clone H4A3, Becton Dickinson), 3μl 7-AAD (Becton Dickinson) as viability marker and 0.2μl mouse Fc block (BD). The percent of CD63 expressing cells was determined by flow cytometry on a Novocyte Quanteon (Agilent). Histamine levels were determined using an enzyme immunoassay kit (Beckman Coulter).

### Liquid chromatography mass spectrometry (LC/MS)

For phosphoproteomics, 1x10^6^ BMMC per condition were subjected to FcεRI crosslinking for 2 minutes as described above, centrifuged at 4 ⁰C, and pellets were frozen at -80 ⁰C until analysis at IQ Proteomics (Cambridge, MA).

### Cell lysis for proteomics

Cell pellets were lysed in 8 M urea + 100mM EPPS pH 8.0 + 1% sodium dodecyl sulfate (1% w/v) + HALT protease and phosphatase inhibitors (Thermo). Lysates were bead beat at 4^o^C using a Precellys Evolution homogenizer. Protein concentration in the lysates was quantified using the Pierce micro BCA assay (Thermo). Proteins were reduced with 5 mM DTT and alkylated with 15 mM iodoacetamide. All protein from each sample was precipitated using methanol/chloroform. Following methanol/chloroform precipitation the protein pellet was washed three times with 100% methanol.

### LysC/trypsin digestion, TMT labelling, and pSTY enrichment and fractionation

The dried protein pellets were resuspended in 2 M guanidine-HCl, 100 mM EPPS, pH 8.0. Approximately 0.5 mg protein per sample was digested with LysC (Wako Chemicals) at a 1:25; protease:protein ratio and the proteins were digested at 25°C for 12 h. Following the LysC digestion, the guanidine-HCl concentration was diluted to 0.5 M with 100 mM EPPS, pH 8.0 and trypsin (Promega, Madison, WI) was added at 1:50; protease:protein ratio. Trypsin digestion was carried out at 37°C for 8 h. Peptide concentrations were quantified using the Pierce peptide fluorescence assay (Thermo). 0.5 mg of peptide per sample was labelled with TMT 11-plex reagent (Thermo) at a 3:1 ratio (TMT reagent:peptide), incubating at 25°C for 3h. TMT reactions were quenched with 0.5% hydroxylamine and samples were acidified with TFA and combined into a “final mix”. The final mix peptides were desalted on 200 mg Waters tC18 SepPak cartridges (Waters Corporation, Milford, MA) and dried by centrifugal evaporation. pY phosphopeptides were enriched using the Cell Signaling Technologies pY-1000 antibody kit as per the manufacturers protocol. pY enriched peptides were desalted on Empore-C18 (3M) in-house packed StageTips prior to analysis by mass spectrometry. pY peptides were reconstituted in 5% formic acid + 5% acetonitrile for LC-MS3 analysis. The flow through from the pY enrichment was desalted on 200 mg Waters tC18 SepPak cartridges and dried by centrifugal evaporation. pST phosphopeptides were enriched using the Pierce Fe-NTA phospho-enrichment kit (Thermo). In brief, the dried peptides from the pY flow through were resuspended in binding buffer provided with the kit. The peptides were bound and washed as per manufacturers protocol. Phosphopeptides were eluted from the Fe-NTA resin with 50mM HK2PO4 pH 10.5. Labelled Phosphopeptides were fractionated using the high pH reverse-phase peptide fractionation kit (Pierce) into 3 fractions (10%, 13%, 15%, 18%, 25%, and 50% acetonitrile in 0.1% triethylamine) and desalted with Empore-C18 (3M) in-house packed StageTips prior to analysis by mass spectrometry. The samples were resuspended in 0.1% TFA desalted on StageTips and vacuum dried. pST peptides were reconstituted in 5% formic acid + 5% acetonitrile for LC-MS3 analysis.

### Mass spectrometry analysis

All mass spectra were acquired on an Orbitrap Fusion Lumos coupled to an EASY nanoLC-1200 (Thermo) liquid chromatography system. Approximately 2 μg of peptides were loaded on a 75 μm capillary column packed in-house with Sepax GP-C18 resin (1.8 μm, 150 Å, Sepax) to a final length of 35 cm. pST peptides were separated using a 120 minute linear gradient from 5% to 23% acetonitrile in 0.1% formic acid. The mass spectrometer was operated in a data dependent mode. The scan sequence began with FTMS1 spectra (resolution = 120,000; mass range of 350-1400 m/z; max injection time of 50 ms; AGC target of 1e6; dynamic exclusion for 60 seconds with a +/- 10 ppm window). The ten most intense precursor ions were selected for ITMS2 analysis via collisional-induced dissociation (CID) in the ion trap (normalized collision energy (NCE) = 35; max injection time = 200ms; isolation window of 0.7 Da; AGC target of 2e4). Following MS2 acquisition, a synchronous-precursor-selection (SPS) MS3 method was enabled to select five MS2 product ions for high energy collisional-induced dissociation (HCD) with analysis in the Orbitrap (NCE = 55; resolution = 50,000; max injection time = 300 ms; AGC target of 1e5; isolation window at 1.2 Da for +2 m/z, 1.0 Da for +3 m/z or 0.8 Da for +4 to +6 m/z). pY peptides were separated using a 180 min linear gradient from 7% to 26% acetonitrile in 0.1% formic acid. The mass spectrometer was operated in a data dependent mode. The scan sequence began with FTMS1 spectra (resolution = 120,000; mass range of 350-1400 m/z; max injection time of 50 ms; AGC target of 1e6; dynamic exclusion for 75 seconds with a +/- 10 ppm window). The ten most intense precursor ions were selected for FTMS2 analysis via collisional-induced dissociation (CID) in the ion trap (normalized collision energy (NCE) = 35; max injection time = 150ms; isolation window of 0.7 Da; AGC target of 3e4; m/z = 2-6; Orbitrap resolution = 15k). Following FTMS2 acquisition, a synchronous-precursor-selection (SPS) MS3 method was enabled to select five MS2 product ions for high energy collisional-induced dissociation (HCD) with analysis in the Orbitrap (NCE = 55; resolution = 50,000; max injection time = 300 ms; AGC target of 1e5; isolation window at 1.2 Da. All mass spectra were converted to mzXML using a modified version of ReAdW.exe. MS/MS spectra were searched against a concatenated 2018 human Uniprot protein database containing common contaminants (forward + reverse sequences) using the SEQUEST algorithm (2). Database search criteria are as follows: fully tryptic with two missed cleavages; a precursor mass tolerance of 50 ppm and a fragment ion tolerance of 1 Da for peptide and phosphoserine and phosphothreonine (phosphotyrosine fragment ion tolerances were set to 0.02 Da); oxidation of methionine (15.9949 Da) or pSTY (79.9663304) was set as differential modifications. Static modifications were carboxyamidomethylation on cysteines (57.02146374) and TMT on lysines and N-termini of peptides (229.162932). Peptide-spectrum matches were filtered using linear discriminant analysis (3) and adjusted to a 1% peptide false discovery rate (FDR) (4) and collapsed further to a final 1.0% protein-level FDR. Posttranslational modifications were localized using a probability-based algorithm similar to Ascore (5). Proteins were quantified by summing the total reporter intensities across all matching PSMs.

### Proteomics data analysis

Differential peptide phosphorylation between sample groups was computed by moderated t-test using the limma r package (6) on the log2 transformed peptide intensities. Phosphopeptides with false discovery rate (FDR) <0.05 (7) were considered significant. Heatmap visualizations of the scaled peptide intensities were plotted by ComplexHeatmap package (8). Phosphopeptides increasing or decreasing by at least 20%, between FcεRI stimulated and unstimulated cells, and with a FDR<0.05 were retained for the kinase analysis. Similarly, phosphopeptides increasing or decreasing by at least 50%, between isotype FcεRI stimulated cells and Siglec-8 FcεRI stimulated cells, and with a FDR<0.1, were retained for the kinase tree visualization. Mouse kinase-substrate annotation was retrieved from PhosphositePlus (9) by PhosR package (10). Visualization of the kinase tree map was obtained with KinMap (11).

### Intracellular flow cytometry

After treatment cells were fixed by adding PFA to a final concentration 3% and incubation at room temperature for 10 min, then washed in FACS buffer and permeabilized by resuspending in cold (-20 ⁰C) methanol, added slowly while shaking, then incubated on ice for 30 min and stained with anti-p-Syk-AF488 (Tyr525/526, Cell Signaling Technology 4349) and anti-p-Erk-PE (Thr202/Tyr204, CST 14095). Cells were washed in FACS buffer and analyzed by flow cytometry.

### IgE treatment of BMMCs

Mouse IgE antibody (clone SPE-7, Millipore Sigma) was added at the indicated concentrations to S8-BMMC 2 hrs prior to FcεRI-mediated activation as described in the main methods section.

## Supplementary References

1. Saito H, Kato A, Matsumoto K, Okayama Y. Culture of human mast cells from peripheral blood progenitors. Nat Protoc. 2006;1(4):2178-83.

2. Eng JK, McCormack AL, Yates JR. An approach to correlate tandem mass spectral data of peptides with amino acid sequences in a protein database. J Am Soc Mass Spectrom. 1994;5(11):976-89.

3. Huttlin EL, Jedrychowski MP, Elias JE, Goswami T, Rad R, Beausoleil SA, et al. A tissue-specific atlas of mouse protein phosphorylation and expression. Cell. 2010;143(7):1174-89.

4. Elias JE, Gygi SP. Target-decoy search strategy for increased confidence in large-scale protein identifications by mass spectrometry. Nat Methods. 2007;4(3):207-14.

5. Beausoleil SA, Villen J, Gerber SA, Rush J, Gygi SP. A probability-based approach for high-throughput protein phosphorylation analysis and site localization. Nat Biotechnol. 2006;24(10):1285-92.

6. Ritchie ME, Phipson B, Wu D, Hu Y, Law CW, Shi W, et al. limma powers differential expression analyses for RNA-sequencing and microarray studies. Nucleic Acids Res. 2015;43(7):e47.

7. Benjamini Y, Hochberg Y. Controlling the false discovery rate: a practical and powerful approach to multiple testing. Journal of the Royal Statistical Society. 1995;57(1):289-300.

8. Gu Z, Eils R, Schlesner M. Complex heatmaps reveal patterns and correlations in multidimensional genomic data. Bioinformatics. 2016;32(18):2847-9.

9. Hornbeck PV, Zhang B, Murray B, Kornhauser JM, Latham V, Skrzypek E. PhosphoSitePlus, 2014: mutations, PTMs and recalibrations. Nucleic Acids Res. 2015;43(Database issue):D512-20.

10. Kim HJ, Kim T, Hoffman NJ, Xiao D, James DE, Humphrey SJ, et al. PhosR enables processing and functional analysis of phosphoproteomic data. Cell Rep. 2021;34(8):108771.

11. Eid S, Turk S, Volkamer A, Rippmann F, Fulle S. KinMap: a web-based tool for interactive navigation through human kinome data. BMC Bioinformatics. 2017;18(1):16.

## Supplementary Data

### Proteomics data spreadsheet

See Spreadsheet

# Supplementary Figures and Tables

## Supplementary Figures


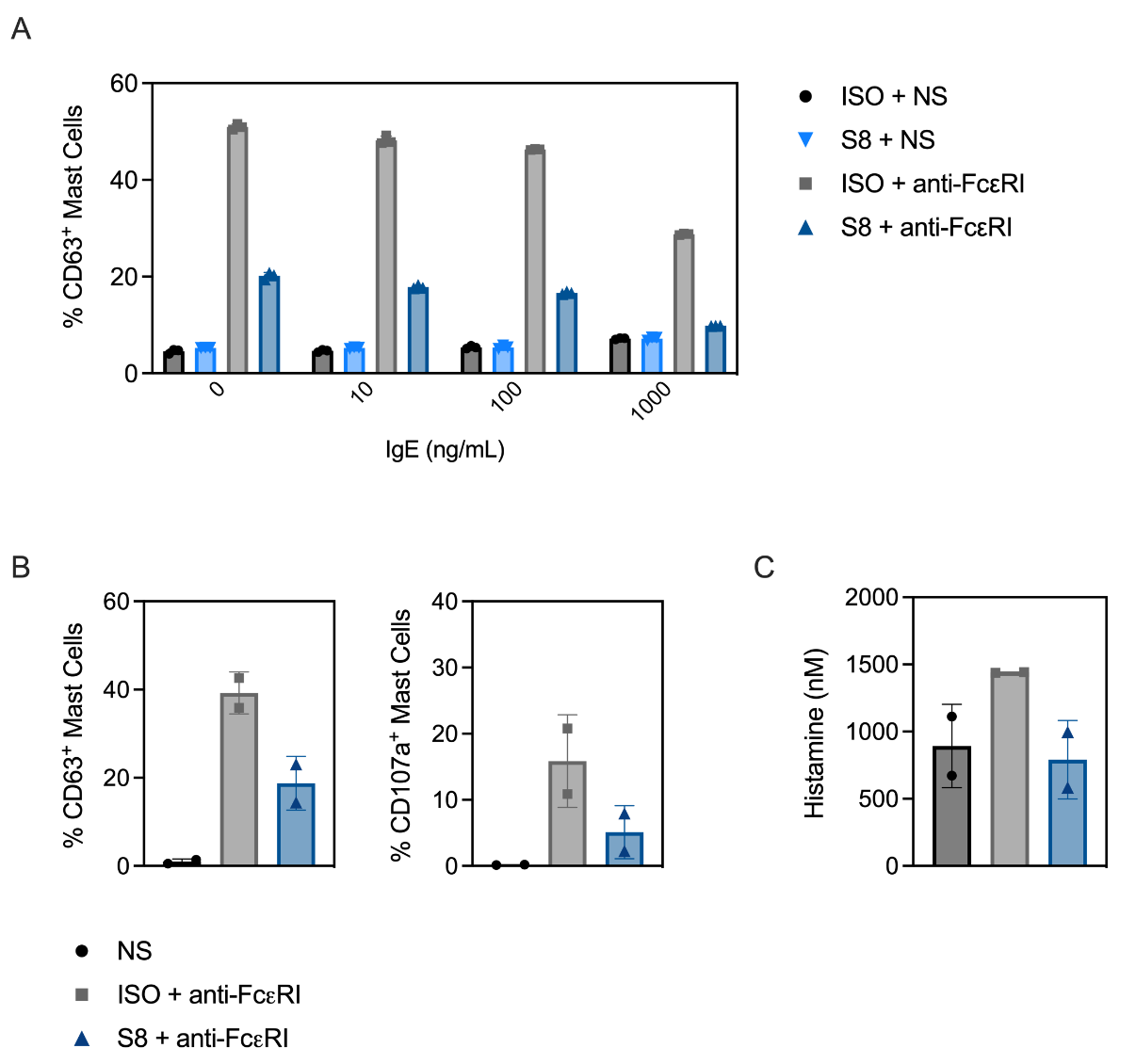


**Supplementary Figure 1. Siglec-8 mediated inhibition of human primary CD34+ cell derived MC from 2 donors of FcεRI-mediated activation. (A)** Percent of CD63 positive MC in unstimulated (ISO + NS, S8 + NS), stimulated (ISO + anti-FcεRI) and inhibited (S8 + anti-FcεRI) MC after pre-incubation with the indicated concentrations of IgE for 2 hours. **(B)** Levels of surrogate degranulation markers CD63 and CD107a on the cell surface of unstimulated MC (black bars), when cross-linked with the anti-FcεRI antibody CRA-1 for 15 min (grey bars) or co-cross-linked with CRA-1 and Siglec-8 mAb (blue bars). **(C)** Histamine concentrations in the culture medium of MC collected 1 hour after stimulation as in (B). NS=unstimulated, S8=Siglec-8, ISO=isotype


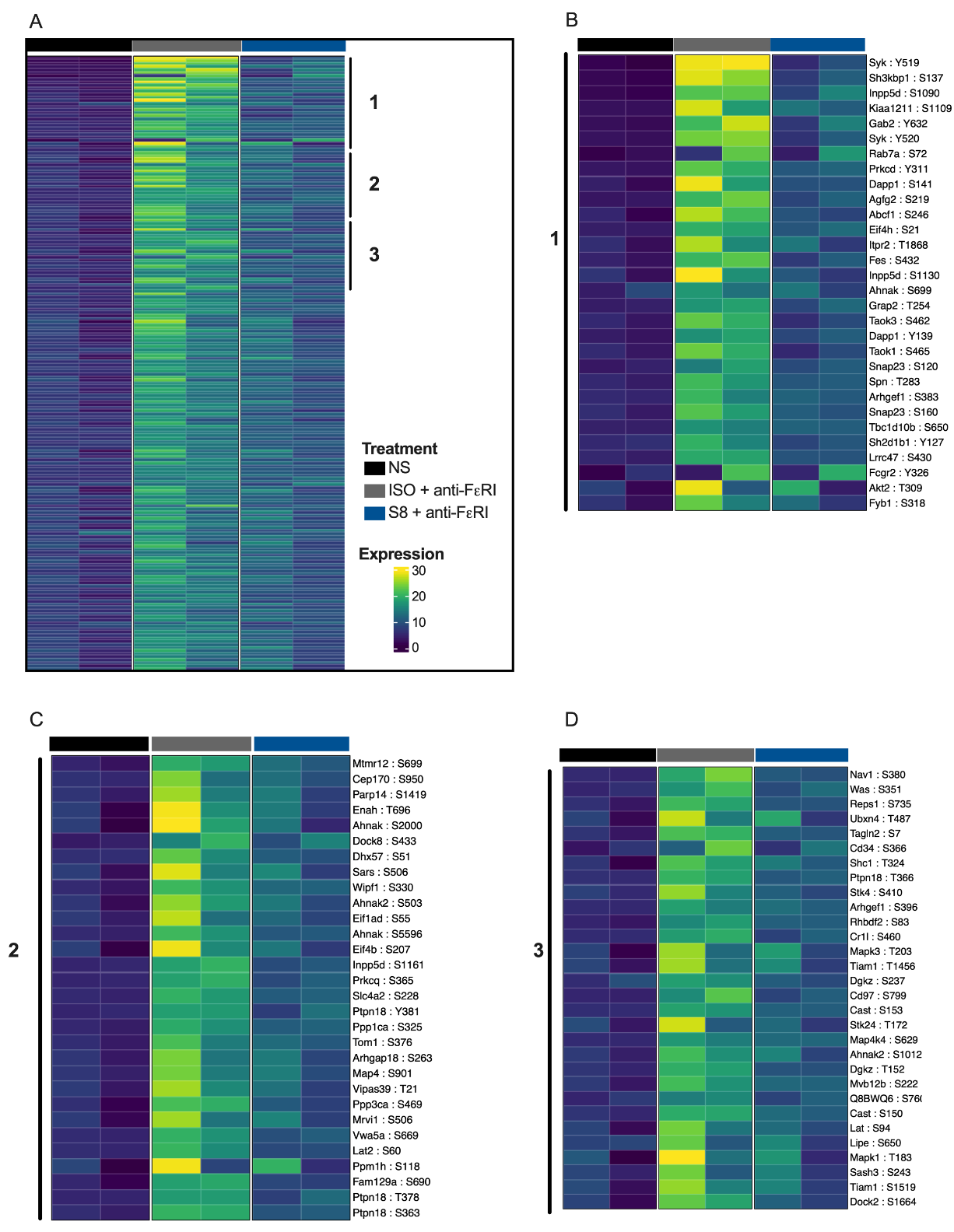


**Supplementary Figure 2. Heatmaps of proteomics data. (A)** Heatmap of quantified phospho-peptides. Each line represents a unique phospho-peptide with the first two columns representing the unstimulated MCs in two independent experiments, the next two columns the phospho-proteome from MCs activated through FcεRI and the last two columns represent activation in the presence of a Siglec-8 mAb. The heatmap is ranked from top to bottom by fold induction upon activation averaged over the two experiments. **(B)** Zoom in on top 30 quantified peptides form heatmap in A. **(C)** Peptides 31-60 from heatmap in A. **(D)** Peptides 61-90 from heatmap in A.


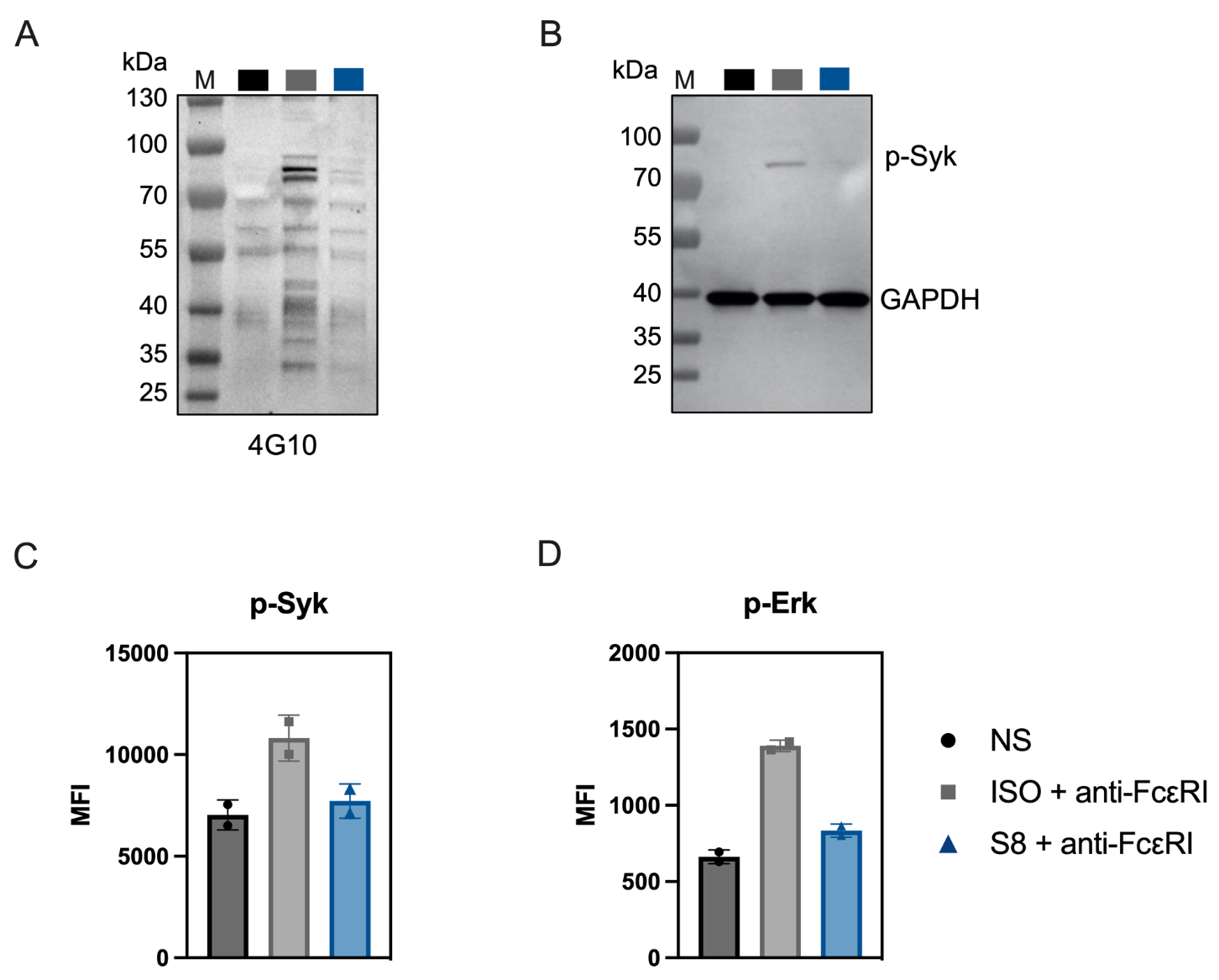


**Supplementary Figure 3.** **Confirmation of induced and inhibited phosphorylation events in MCs treated as described in Figure 3.** **(A)** Phospho-tyrosine western blot analysis of whole cell lysates. black=NS, gray=ISO + anti-FcεRI, blue= S8 + anti-FcεRI **(B)** p-Syk western blot analysis of whole cell lysates. **(C)** Flow cytometry analysis of intracellular phospho-Syk. **(D)** Flow cytometry analysis of intracellular phospho-Erk. NS=unstimulated, S8=Siglec-8, ISO=isotype
